# Stable Mutated tau441 Transfected SH-SY5Y Cells as Screening Tool for Alzheimer’s Disease Drug Candidates

**DOI:** 10.1007/s12031-012-9716-6

**Published:** 2012-02-19

**Authors:** Tina Löffler, Stefanie Flunkert, Nicole Taub, Emma L. Schofield, Malcolm A. Ward, Manfred Windisch, Birgit Hutter-Paier

**Affiliations:** 1 JSW Life Sciences GmbH, Parkring 12, 8074 Grambach, Austria; 2 Proteome Sciences plc, London, UK

**Keywords:** Tau, Hyperphosphorylation, Kinase inhibitor, Drug development

## Abstract

The role of hyperphosphorylation of the microtubule-associated protein tau in the pathological processes of several neurodegenerative diseases is becoming better understood. Consequently, development of new compounds capable of preventing tau hyperphosphorylation is an increasingly hot topic. For this reason, dependable *in vitro* and *in vivo* models that reflect tau hyperphosphorylation in human diseases are needed. In this study, we generated and validated an *in vitro* model appropriate to test potential curative and preventive compound effects on tau phosphorylation. For this purpose, a stably transfected SH-SY5Y cell line was constructed over-expressing mutant human tau441 (SH-SY5Y-TMHT441). Analyses of expression levels and tau phosphorylation status in untreated cells confirmed relevance to human diseases. Subsequently, the effect of different established kinase inhibitors on tau phosphorylation (e.g., residues Thr231, Thr181, and Ser396) was examined. It was shown with several methods including immunosorbent assays and mass spectrometry that the phosphorylation pattern of tau in SH-SY5Y-TMHT441 cells can be reliably modulated by these compounds, specifically targeting JNK, GSK-3, CDK1/5, and CK1. These four protein kinases are known to be involved in *in vivo* tau phosphorylation and are therefore authentic indicators for the suitability of this new cell culture model for tauopathies.

## Introduction

The microtubule-associated protein tau is an abundantly represented protein in the central and peripheral nervous system, especially concentrated in axons of neurons (Goedert 1996). The most studied function of tau is its binding to microtubules and promotion of microtubule assembly and stabilization (Trinczek et al. 1995; Wang and Liu 2008). In tauophathies, like Alzheimer’s disease (AD), progressive supranuclear palsy (PSP), frontotemporal dementia and Parkinsonism linked to chromosome 17 (FTDP), corticobasal degeneration, and frontotemporal dementia, tau protein is hyperphosphorylated causing its aggregation, and thereby weakening its microtubule-stabilizing effect. For a long time, it was thought that tau aggregates affect neurotoxicity and are therefore the main cause of tauopathy-related neurodegeneration and dementia. However, new research shows that hyperphosphorylated rather than insoluble aggregated tau seems to initiate neuronal death and memory deficits (SantaCruz et al. 2005; de Calignon et al. 2010; Feuillette et al. 2010; Fox et al. 2011). In particular, it has recently been shown that soluble hyperphosphorylated tau protein disrupts normal synaptic transmission in *Drosophila* (Cowan et al. 2010), mouse (Hoover et al. 2010), and giant squid (Moreno et al. 2011) neurons independently of neurodegeneration or loss of synapses, suggesting that this is an early event in the evolution of cognitive impairment associated with tauopathies. Pharmacological interventions of tau phosphorylation are thought to present a new avenue in the treatment of tauopathies (Mazanetz and Fischer 2007; Hanger et al. 2009; Gozes 2011; Shiryaev et al. 2011). Thus, GSK-3 inhibitors are already in clinical trials for the treatment of various disorders (Medina and Castro 2008), indicating that there is an urgent need for *in vitro* and *in vivo* systems, including cell culture models mimicking tauopathies and other phosphorylation-related diseases to accelerate development of new active molecules. For this purpose, we generated a stable transfected SH-SY5Y cell line expressing human tau441 comprising two mutations, V337M and R406W (Hasegawa et al. 1997; Hutton et al. 1998; Nacharaju et al. 1999). Both mutations transfected separately are already shown to induce phosphorylation of tau at residues Thr231 and Ser396, Ser406, respectively (Yanagi et al. 2009). To analyze the tau expression efficacy in this cell line, we verified the phosphorylation status of tau at residues Thr181, Ser202, Thr231, and Ser396. These phosphorylation sites are all known to influence the binding and/or the stabilization properties of tau to microtubules and thus supporting disease-related features of tau (Bramblett et al. 1993; Cho and Johnson 2004; Han et al. 2009). In order to use the SH-SY5Y-TMHT441 cell line for compound screening or lead optimization, it is necessary to provide quantitative methods for measuring multiple phosphorylation events on tau protein to confirm that the phosphorylation status of different sites is modulated by different kinase inhibitors. While a number of immunological methods for measuring tau phosphorylation are available, they are limited in the number of sites covered and often cannot distinguish between closely related sites. The mass spectrometry-based technique Selected Reaction Monitoring (SRM) is becoming widespread in the validation and routine measurement of protein biomarkers (Lange et al. 2008), and as such, assays are being developed and applied in CNS disorders (Lopez et al. 2011). SRM enables the site-specific monitoring of multiple individual phosphorylation sites that may be closely related in a single assay, as well as monitoring single amino acid mutations such as R406W. Consequently, we treated the cells with JNK, GSK-3, CDK1/5, or CK-I inhibitors since these protein kinases are well known to be involved in the phosphorylation of tau (Hanger et al. 2009) and measured tau phosphorylation status with both immunological and SRM methods. Our results demonstrate the flexibility of the established cellular model and tau phosphorylation assays and the close similarities between the SH-SY5Y-TMHT441 cell line *in vitro* and *in vivo* results (Flunkert et al., unpublished data). Characterization of this *in vivo* TMHT mouse model revealed increasing soluble, but not insoluble total tau and ptau (Thr231) levels over age and increased human ptau at residues Thr181, Ser199, Thr231, and Thr235. Furthermore, the TMHT mouse model showed a progressive increase in human tau protein in the amygdala over age and strong spatial learning deficits as early as 5 months of age as well as olfactoric deficits.

In summary, we have established a new *in vitro* system comprising a novel stable cell line and phosphorylation site assays that is applicable for medium throughput screening of early drug candidates modulating tau phosphorylation, selected for pre-clinical development. The combination of stable transgenic cell lines for tau with bespoke mass spectrometry assays opens a new window of opportunity to successfully combat tauopathies.

## Materials and Methods

### Cell Line

SH-SY5Y cells, a clone of the human neuroblastoma cell line SK-N-SH, were purchased from LGC Standards. Cells were cultured in DMEM (Lonza) supplemented with 10% fetal calf serum (Lonza), 200 mM l-glutamine (Lonza), non-essential amino acids (NEAA, 100×) (HyClone), and 10 mg/ml gentamycin (Invitrogen) at 37°C and 5% CO_2_ in a humidified atmosphere.

### Stable Transfection of SH-SY5Y Cells

For stable transfection of SH-SY5Y cells, a pcDNA3-TMHT441 (V337M/R406W) construct was used. The TMHT441 gene contains the longest isoform of human tau regulated by a CMV promoter and carries two well-characterized mutations known to promote tau aggregation and/or hyperphosphorylation. The construct also contains an ampicillin and neomycin resistance gene. Before stable transfection, cells were seeded in six-well plates at a density of 5 × 10^5^ per well. Upon 24 h, cells were transfected with FugeneHD transfection reagent (Roche) according to the manufacturer’s protocol. After 24 h incubation, the culture medium was changed. Following another 24 h, cells were transferred to 10 cm dishes in selective medium containing 300 μg/ml gentamycin-disulfate G418 (Roth) and 1 × 10^6^ untransfected SH-SY5Y cells as feeder cells that died after a few days on the selective medium. After 3 weeks of G418 selection, single clones were isolated and cultured in 96-well plates. Only one clone showed TMHT441 expression. Stable TMHT transfected SH-SY5Y cells were named SH-SY5Y-TMHT441 cells.

### Immunofluorescence Microscopy

For immunofluorescent labeling, SH-SY5Y-TMHT441 cells were seeded on Poly-d-Lysine-coated chamber slides. After 2 days in culture, cells were washed, fixed in 4% paraformaldehyde for 20 min, permeabilized with 0.2% Triton/PBS for 30 min, blocked with 20% horse serum/0.2% BSA/0.1% Triton/PBS for 1 h, and labeled with primary antibody HT7 (Pierce Endogen) in 0.2% BSA/0.05% Triton/PBS for 1 h followed by blocking with 20% horse serum/0.2% BSA/0.1% Triton/PBS for 20 min. Cells were then labeled with secondary fluorescent antibody Cy3 (Jackson ImmunoResearch) in 0.2% BSA/0.05% Triton/PBS for 1 h. DAPI solution (25 mg/ml in A. bidest.) was added 1:2,000 to the secondary antibody solution. After primary and secondary antibody incubations, cells were washed three times with 0.05% Triton/PBS. Samples were mounted with Mowiol and analyzed by fluorescent microscopy.

### Treatment of Cells

For all phosphorylation-related experiments, differentiated SH-SY5Y-TMHT441 cells were used. Therefore, cells were treated with 10 μM retinoic acid for 7 days. Differentiated cells were treated with 0.1–50 μM kinase inhibitors SP600125 (Sigma), AR-A014418 (Sigma), RP106 (Aloisine; Calbiochem), IC261 (Calbiochem), or 0.2% DMSO as vehicle for 4 h. Each kinase inhibitor was tested in two autonomous experiments, and in each case cell viability was determined using a standard MTT assay (Promega).

After treatment, cells were washed twice with cold PBS and harvested for Western blotting, Mesoscale Discovery, and mass spectrometry analyses. For all measurements, cells were harvested at a confluency of 90% in RIPA buffer, containing 1× protease inhibitor cocktail (Calbiochem) as well as 1× phosphatase inhibitor cocktail 3 (Sigma) and sonicated on ice. Total protein amount of lysates was determined with a BCA Protein Assay Kit (Pierce Endogen). For Western blotting, the following antibodies were used: Tau-5, β-actin (Abcam); AD2 (BioRad), AT270, AT180, AT8 (Thermo Scientific).

Total tau and phosphorylated tau at residues Thr181, Thr231, and Ser396 of vehicle only and inhibitor treated SH-SY5Y-TMHT441 cells were analyzed by Multi Array® Phospho-Tau (Thr 231)/Total Tau immunosorbent Assay and two prototype assays, Phospho-Tau (Thr181) and Phospho-Tau (Ser396), (MSD; mesoscale discovery).

To increase the range of individual sites covered by precise quantitative assays, we adopted two SRM-based assays (Phospho-Tau SRM assay versions 2 and 2.1; Proteome Sciences plc, London, UK) (Schofield et al. 2011). The version 2 assay measures the extent of phosphorylation at Thr181, Ser199, Thr231, Ser262, and Ser396 (human 2N4R tau numbering). The version 2.1 assay was developed specifically for tau carrying the R406W mutation which lacks a trypsin cleavage site at R406 necessary for the version 2 assay. Consequently, the 2.1 assay measures phosphorylation at Ser396 and Ser404 in the presence of the R406W mutation, where residue 406 is changed from R to W. Both assays use a triple quadrupole mass spectrometer to monitor selective fragments of precursor ions from phosphopeptides produced by enzymatic digestion of tau. Each precursor ion is isolated, fragmented, and then selected fragment ions are quantified against known amounts of heavy labeled phosphopeptide standards.

### Mice

Brain tissue of TMHT (Thy-1 Mutated Human Tau) transgenic mice over-expressing the longest human tau isoform tau441 (2N4R) carrying two mutations, V337M and R406W, under control of the neuron specific murine Thy-1 (mThy-1) promoter was used as positive control (Flunkert et al., unpublished data).

### Protein Extraction from Murine Tissue

Protein was extracted from the hippocampus and cortex as described previously (Delobel et al. 2008) with slight modifications. In brief, tissues were homogenized in 100 μl cold extraction buffer (25 mM Tris–HCl, pH 7.4, 150 mM NaCl, 1 mM EDTA, 1 mM EGTA, 10 mM β-glycerophosphate, 30 mM sodium fluoride, 2 mM sodium orthovanadate). Protease (Calbiochem) and phosphatase (Sigma) inhibitors were added before use. The homogenates were spun for 15 min at 74,200 × *g* and the supernatants were used for the analysis of tau. Immunoblots were performed with cortical and hippocampal protein lysates of 12-month-old hemizygous TMHT transgenic and corresponding non-transgenic brain tissue. For a detailed characterization of the TMHT transgenic mouse model, see Flunkert et al. (unpublished data).

### Statistical Analyses

All data received from immunosorbent assay (MSD) were analyzed using unpaired *t* test or one-way ANOVA followed by Dunett’s *post hoc* test. All statistical analyses were performed with Graph Pad Prism4, version 4.03. Data received from SRM assay were not statistically analyzed since group size (*n* = 1) was too small.

## Results

**Fig. 1:**
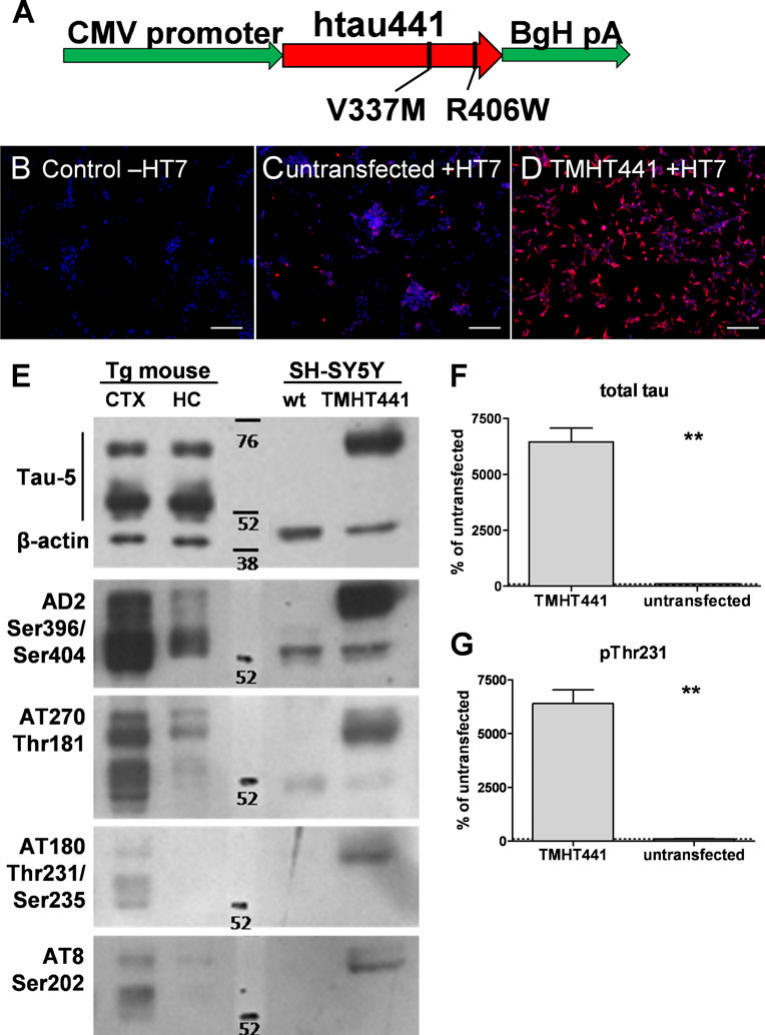
Characterization of TMHT expression in SH-SY5Y-TMHT441 cells and in brain tissue from TMHT mice. **a** Schematic presentation of the tau441 construct with two mutations (TMHT). **b–d** SH-SY5Y-TMHT441 cells were subjected to indirect immunofluorescence analysis using antibodies against HT7 (tau, *red*) and DAPI (nuclei, *blue*). Images show secondary antibody control (**b**), untransfected SH-SY5Y cells (**c**), and SH-SY5Y-TMHT441 cells (**d**). *Scale bar*: 100 μm. **e** Western blot of SH-SY5Y-TMHT441 cells compared to untransfected cells (*wt*) and cortical (*CTX*) and hippocampal (*HC*) brain tissue homogenates of 12-month-old TMHT transgenic mice. Antibody Tau-5 labels human and mouse tau protein. AD2, AT270, AT180, and AT8 antibodies detect Ser396/Ser404, Thr181, Thr231/Ser235, and Ser202 phosphorylation, respectively. A representative blot of two independent experiments is shown. **f**, **g** Measurement of total tau (**f**) and pThr231 (**g**) levels in untransfected and stable TMHT441 transfected cells by Mesoscale Discovery (*N* = 2). **P < 0.01; unpaired *t* test. *Graphs* represent mean ± SEM

Recently, we demonstrated that mice over-expressing tau441 with V337M/R406W mutations serve as an appropriate animal model for drug candidate testing (Flunkert et al., unpublished data). To allow pre-screening of drug candidates and prioritization for transfer into advanced pre-clinical development, we established an *in vitro* AD model by stably transfecting SH-SY5Y cells with the TMHT441 construct which has already been used successfully for the generation of an *in vivo* model (Fig. 1a), later referred to as SH-SY5Y-TMHT441 cells.

### SH-SY5Y-TMHT441 Cells Express High Levels of Total Tau that is Highly Phosphorylated at Different Sites

To determine the expression levels of the TMHT construct, SH-SY5Y-TMHT441 cells were labeled with a human specific anti-tau antibody (HT7) by immunofluorescence staining. All SH-SY5Y-TMHT441 cells strongly over-express the TMHT construct compared to non-transfected control SH-SY5Y cells (Fig. 1c vs. 1d), in which endogenous tau expression was low (Fig. 1c). To validate antibody specificity, control SH-SY5Y cells were incubated with secondary antibody only (Fig. 1b) and no signal was detected. To quantitatively analyze TMHT expression and phosphorylation, Western blots and Mesoscale Discovery analyses of SH-SY5Y-TMHT441 and non-transfected SH-SY5Y cells were performed (Fig. 1e and f–g, respectively). For Western blots, cortical and hippocampal brain tissue from TMHT transgenic mice served as positive controls. Thus, SH-SY5Y-TMHT441 cells express transgenic human tau slightly stronger than TMHT mice which also express endogenous mouse tau, as shown by Tau-5 Western blots. Analyses of the phosphorylation status of SH-SY5Y-TMHT441 cells revealed high levels of ptau at residue Ser396/Ser404, Thr181, and slightly lower levels at Thr231/Ser235 and Ser202 (Fig. 1e). Thus, immunoblot analyses confirmed a comparable phosphorylation status of cortical tissue of TMHT mice versus SH-SY5Y-TMHT441 cells. Analyses of total and ptau at residue Thr231 by Mesoscale Discovery showed that both total and ptau Thr231 levels in SH-SY5Y-TMHT441 cells are about 60-fold higher compared to SH-SY5Y cells (Fig. 1f, g). The ratio of total and ptau Thr231 stays the same in SH-SY5Y-TMHT441 and SH-SY5Y cells.

Next, we analyzed whether different kinases, including JNK, GSK3-β, Cdk1/5, and CK1 kinase, which are known to contribute to tau phosphorylation, are also involved in tau phosphorylation of SH-SY5Y-TMHT441 cells. In all experiments, two negative controls were used, untreated and vehicle treated SH-SY5Y-TMHT441 cells, and all presented data were normalized to the corresponding vehicle control. Additionally, normalization of data to cell toxicity did not change the outcome of the assay (data not shown).

### SP600125 Strongly Inhibits pThr181 and pThr231 Tau Phosphorylation

**Fig. 2:**
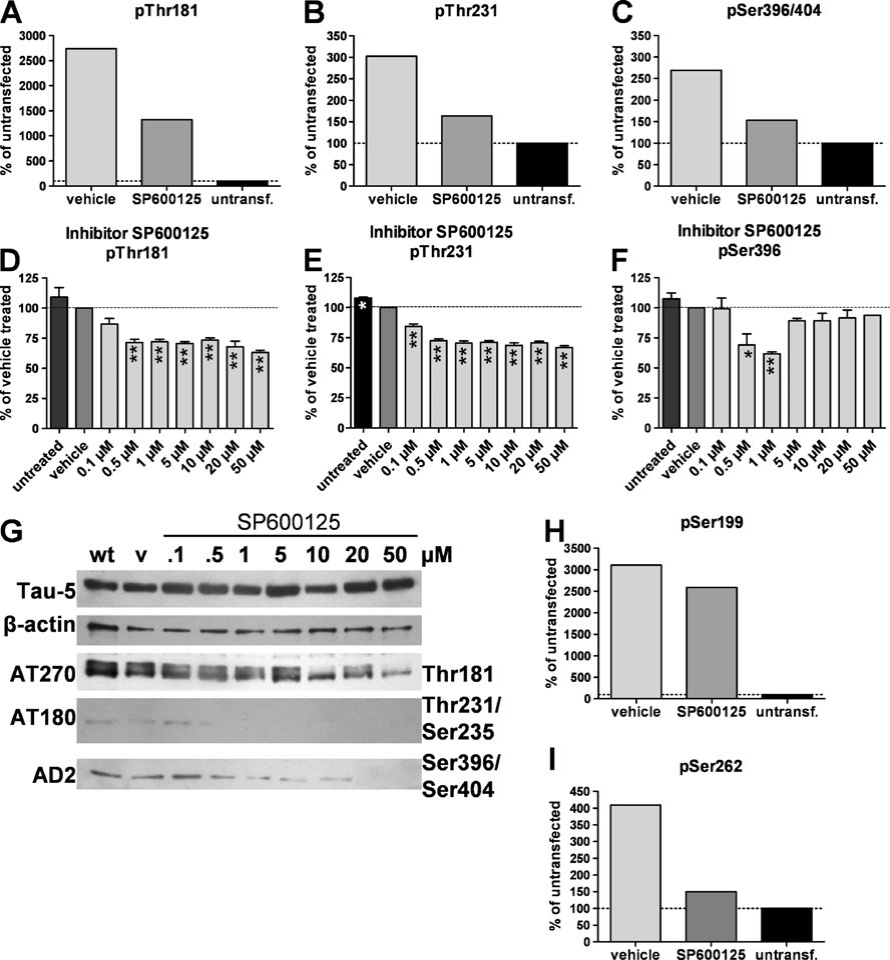
JNK inhibitor SP600125 strongly inhibits tau phosphorylation at residue Thr181, Thr231, and Ser199 and to a lesser extent Ser396 in SH-SY5Y-TMHT441 cells. **a–c** Effect of JNK inhibitor SP600125 (50 μM) on Thr181 (**a**), Thr231 (**b**), and Ser396 (**c**) phosphorylation as measured by Phospho-Tau SRM assay (*N* = 1). **d–f** Effect of JNK inhibitor SP600125 on Thr181 (**d**), Thr231 (**e**), and Ser396 (**f**) phosphorylation as measured by Mesoscale Discovery. First lane (*black*) shows protein of untreated cells, second lane (*dark gray*) protein of vehicle treated cells and *gray bars* show SP600125 treated cells (*N* = 2). *P < 0.05, **P < 0.01. *Graphs* represent mean ± SEM. One-way ANOVA followed by Dunnett’s *post hoc* test. **g** Western Blot of untreated (*wt*), vehicle (*v*) treated, and SP600125 (concentration ranging from 0.1–50 μM) treated SH-SY5Y-TMHT441 cells showing total tau (*Tau-5*), β-actin as loading control, pThr181 (*AT270*), pThr231/235 (*AT180*), and pSer396 (*AD2*) levels. A representative blot of two independent experiments is shown. **h** Effect of JNK inhibitor SP600125 on Ser199 as measured by Phospho-Tau SRM assay (*N* = 1). **i** Effect of JNK inhibitor SP600125 on Ser262 as measured by Phospho-Tau SRM assay (*N* = 1)

Differentiated SH-SY5Y-TMHT441 cells were first treated with JNK inhibitor SP600125 or vehicle. Phospho-Tau SRM analyses showed a strong decrease of tau phosphorylation at residues Thr181 and Thr231 when using 50 μM SP600125 (Fig. 2a, b). A strong concentration-dependent decrease of tau phosphorylation at residues Thr181 and Thr231 was measured by Mesoscale Discovery analyses in SP600125 treated cells as compared to vehicle treated control cells (Fig. 2d, e). Analysis of these data by non-linear regression provides IC_50_ values for SP600125 of 100 nM and 91 nM for phosphorylation at Thr181 and Thr231, respectively. Since the applied SP600125 concentrations were in the upper range of the sigmoidal dose response curve (data not shown), the calculated IC_50_ values should be seen as estimates only. Tau phosphorylation at residue Ser396 was almost reduced to half as analyzed by Phospho-Tau SRM when using 50 μM SP600125 and at lower concentrations of 1 μM SP600125 decreased phosphorylation to 62% as measured by Mesoscale Discovery (Fig. 2c, f). At a concentration of 1 μM SP600125, phosphorylation at position Thr181, Thr231, and Ser396 was reduced to 72%, 70%, and 62%, respectively, relative to vehicle treated cells. Additionally, the effect of SP600125 on the phosphorylation status of tau in SH-SY5Y-TMHT441 cells was analyzed by Western blots using phosphorylation-specific antibodies. Immunoblot analysis confirmed the down-regulation of tau phosphorylation at residue Thr181 as obtained from Phospho-Tau SRM and Mesoscale Discovery, although to a lesser extent. Tau phosphorylation at residue Thr231/Ser235 was comparably down-regulated as measured by the other two methods. Performing Western blot analysis with an antibody detecting both phosphorylation at Ser396 as well as Ser404 showed a more distinct down-regulation (Fig. 2g) than Phospho-Tau SRM and Mesoscale Discovery measurements, only detecting phosphorylation at Ser396. Phosphorylation of tau at residue Ser199 was not affected upon SP600125 treatment as determined by Phospho-Tau SRM analysis (Fig. 2h). Additionally, Phospho-Tau SRM analyses also showed a strong decrease of tau phosphorylation at residue Ser262 when using 50 μM SP600125 (Fig. 2i).

### AR-A014418 Strongly Inhibits pSer396 Tau Phosphorylation

**Fig. 3:**
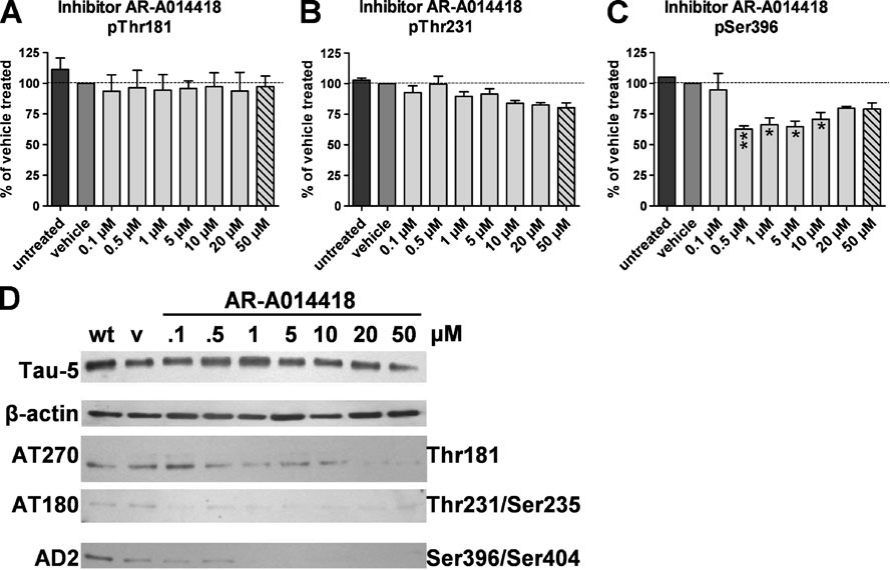
GSK3-β inhibitor AR-A014418 inhibits Thr231 and Ser396 phosphorylation but not Thr181 tau phosphorylation in SH-SY5Y-TMHT441 cells. Effect of GSK3-β inhibitor AR-A014418 on Thr181 (**a**), Thr231 (**b**), and Ser396 (**c**) phosphorylation as measured by Mesoscale Discovery (*N* = 2). First lane (*black*) shows protein of untreated cells. Second lane (*dark gray*) shows protein of vehicle treated cells. *Light gray bars* represent AR-A014418 treated cells; *dashed bars*: treatment caused cell death as measured by MTT assay. *P < 0.05, **P < 0.01. *Graphs* represent mean ± SEM. One-way ANOVA followed by Dunnett’s *post hoc* test. **d** Western blot of untreated (*wt*), vehicle (*v*) treated, and AR-A014418 treated (concentration ranging from 0.1 to 50 μM) SH-SY5Y-TMHT441 cells showing total tau (*Tau-5*), β-actin as loading control, pThr181 (*AT270*), pThr231/235 (*AT180*), and pSer396 (*AD2*) levels. A representative blot of two independent experiments is shown

Next, differentiated SH-SY5Y-TMHT441 cells were treated with the GSK3-β kinase inhibitor AR-A014418 to analyze if GSK3-β kinase is involved in SH-SY5Y-TMHT441 phosphorylation as previously shown in tau transfected 3T3 and CHO cells (Wagner et al. 1996). Analysis of different concentrations of AR-A014418 revealed no effect on phosphorylation at residue Thr181 as determined by Mesoscale Discovery (Fig. 3a); however, a slight concentration-dependent decrease of Thr181 phosphorylation was observed when analyzed by Western blotting (Fig. 3d). AR-A014418 treatment caused a concentration-dependent decrease of phosphorylation at residue Thr231 to 84% when treated with 10 μM AR-A014418, as observed by Mesoscale Discovery (Fig. 3b). Analysis of these data by non-linear regression provides an IC_50_ value for AR-A014418 of 9.1 μM for Thr231. The applied concentrations of AR-A014418 covered the sigmoidal dose–response curve quite well (data not shown). Only lower concentrations of AR-A014418 caused a decrease of Ser396 phosphorylation. When using 10 μM of inhibitor, AR-A014418 phosphorylation at Ser396 was reduced to 71% compared to control samples (Fig. 3c). Western blot analyses of Thr231 and Ser396 partly verified the results of the Mesoscale Discovery analyses (Fig. 3d). The treatment of differentiated SH-SY5Y-TMHT441 cells with 50 μM AR-A014418 was toxic for the cells as verified by MTT assay (data not shown). The corresponding bar graphs therefore represent no valid phosphorylation-modulating effect (Fig. 3a–c, dashed bars; 50 μM).

### RP106 Inhibits pThr181 and pSer396 Tau Phosphorylation Compared to Inhibitor IC261 that Mainly Inhibits pThr231

**Fig. 4:**
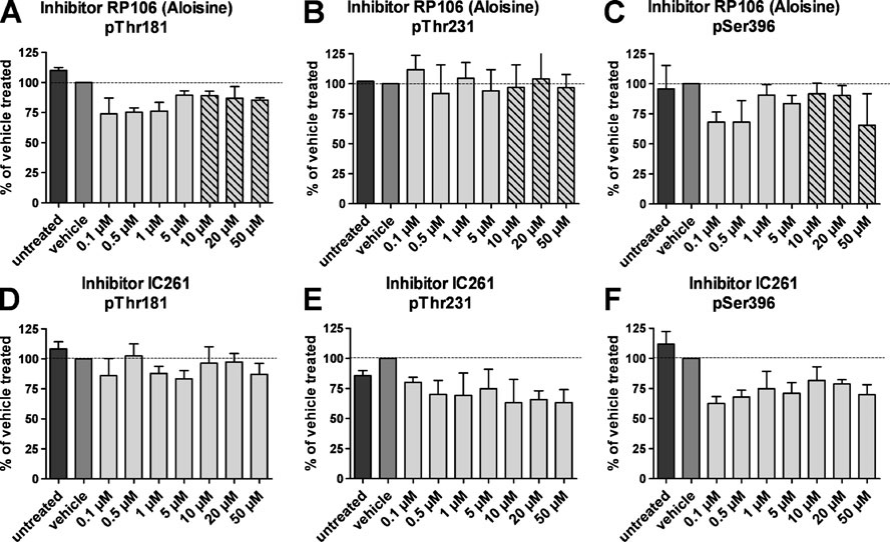
Effect of CDK1/5 and CK-I inhibitor on the phosphorylation status of tau in SH-SY5Y-TMHT441 cells. Effect of CDK1/5 inhibitor RP106 on Thr181 (**a**), Thr231 (**b**), and Ser396 (**c**) phosphorylation as measured by Mesoscale Discovery (*N* =2). Effect of CK-I inhibitor IC261 on Thr181 (**d**), Thr231 (**e**), and Ser396 (**f**) phosphorylation as measured by Mesoscale Discovery (*N*=2). First lane (*black*) shows protein of untreated cells; second lane (*dark gray*) shows protein of vehicle treated cells; *light gray bars* represent inhibitor treated cells; *dashed bars*: treatment caused cell death as measured by MTT assay. *Graphs* represent mean ± SEM. One-way ANOVA followed by Dunnett’s *post hoc* test

Treatment of differentiated SH-SY5Y-TMHT441 cells with increasing concentrations of the Cdk1/5 kinase inhibitor RP106 decreased phosphorylation of tau at residues Thr181 and Ser396 to 74% and 68% at 0.1 μM RP106, respectively, but had no effect on phosphorylation at residue Thr231 as measured by Mesoscale Discovery (Fig. 4a–c). Due to high standard deviations, these differences were not statistically significant. High concentrations of 10–50 μM RP106 were toxic for the SH-SY5Y-TMHT441 cells as verified by MTT assay (data not shown). The corresponding bar graphs represent therefore no valid phosphorylation-modulating effect in this test system (Fig. 4a–c, dashed bars). In contrast, treatment of differentiated SH-SY5Y-TMHT441 cells with increasing concentrations of the CK1 kinase inhibitor IC261 decreased phosphorylation of tau at residues Thr231 and Ser396 to 72% and 68% at 0.5 μM IC261, respectively, but not of Thr181 as analyzed by Mesoscale Discovery (Fig. 4d–f). Analysis of Thr231 phosphorylation data by non-linear regression provides an IC_50_ value of IC261 of 100 nM. Since the used IC261 concentrations were in the upper range of the sigmoidal dose–response curve (data not shown), the calculated IC_50_ value should be seen as estimate only. The CK1 kinase inhibitor consequently caused inhibition of the same phosphorylation sites as the GSK3-β kinase inhibitor.

In summary, our data demonstrate that SH-SY5Y-TMHT441 cells express high levels of human transgenic total tau and phosphorylated tau at residues Thr181, Ser202, Thr231/235, and 396/404. Tau phosphorylation was modifiable by four different kinase inhibitors, suggesting that tau phosphorylation of SH-SY5Y-TMHT441 cells is dependent on the JNK, GSK3-β, Cdk1/5, and CK1 kinase pathway. All these pathways were previously shown to be involved in tau phosphorylation (Hanger et al. 2009) and are therefore a good indicator for the suitability of this new cell culture model for tauopathies.

## Discussion

The aim of the present study was to generate an *in vitro* model of tauopathies that reflects disease relevant tau hyperphosphorylation. For this purpose, SH-SY5Y cells were stably transfected with human TMHT containing two mutations, V337M and R406W. The expression and phosphorylation status of tau was thoroughly analyzed using different biochemical methods. Results indicate that the transgenic cell line displays major biochemical features of human tauopathies and thus is an appropriate tool for early drug screening.

Using immunocytological and biochemical approaches, we first verified if the SH-SY5Y-TMHT441 cells over-express tau protein. Furthermore, we analyzed tau phosphorylation at verified disease relevant residues, including Ser181, Ser199, Thr231, and Ser396 (Hanger et al. 2009). Comparison of SH-SY5Y-TMHT441 cells and TMHT mice revealed a similar tau phosphorylation status between the *in vitro* and *in vivo* models (Flunkert et al., unpublished data). Measurement of total and ptau Thr231 by Mesoscale Discovery confirmed Western blotting results.

The phosphorylation of tau has been the subject of intense research in recent years, and its phosphorylation status in different tauopathies is increasingly well characterized (Ferrer et al. 2005). We thus went on to investigate the contribution of different kinases like JNK, GSK-3, CDK1/5, and CK1, known to be involved in *in vivo* tau phosphorylation (Hanger et al. 2009).

The results of our phosphorylation experiments are summarized in Table 1.

**Table 1:** Tau phosphorylation in TMHT transgenic mice and untreated, SP600125, AR-A014418, RP106, or IC261 treated SH-SY5Y-TMHT441 cells

		SH-SY5Y-TMHT441				
p site	TMHT mice	untreated	SP600125	AR-A014418	RP106	IC261
Thr181	+++	+++	+	+++	++	+++
Ser199	N.A.	+++	++	N.A.	N.A.	N.A.
Thr231	++	++	+	+	++	+
Ser262	N.A.	+++	+	N.A.	N.A.	N.A.
Ser396	+++	++	+	+	++	++

+++ strong phosphorylation, ++ moderate phosphorylation, + weak phosphorylation, *N.A.* not analyzed

Due to mutation R406W, which is close to Ser396, detection and quantification of this phosphorylation site was challenging. Antibodies used for immunoblots as well as Mesoscale Discovery measurements are designed to detect human wild-type tau and are likely to detect mutated tau to a lesser extent. This specific mutation was also shown to indirectly reduce phosphorylation at Ser396 *in vitro* (Tatebayashi et al. 2006), thus leading to a different phosphorylation pattern of mutated and endogenous tau in SH-SY5Y-TMHT441 cells. Especially the differences between actual phosphorylation and detection efficiency of endogenous and mutated tau are possible causes for the lack of concentration dependence of pSer396 reduction in all experiments.

The JNK pathway is of particular interest as JNK proteins are stress induced and activation of JNKs can lead to apoptosis, and hence may contribute to neurodegeneration in AD (Johnson and Nakamura 2007). Inhibition of tau phosphorylation by the JNK inhibitor SP600125 caused decreased phosphorylation at residues Thr181, Thr231, and Ser396 in SH-SY5Y-TMHT441 cells. These results correspond to previous data that demonstrate a contribution of the protein kinase JNK in tau phosphorylation *in vitro* and by co-immunoreactivity of JNK and tau in inclusions of neuronal and glial cells (Reynolds et al. 2000; Atzori et al. 2001). Additionally, Ferrer and colleagues show that JNK is up-regulated in several cell types that also contain pre-tangles or neurofibrillary tangles in brains of familial tauopathy patients (Ferrer et al. 2003). Consequently, our data provide unequivocal evidence for the involvement of JNK in phosphorylation of tau based on *in vitro* and *in vivo* experiments. We did not observe inhibition of tau phosphorylation at residue Ser199 by SP600125, confirming prior analyses that JNK is not required for tau Ser199 phosphorylation (Reynolds et al. 2000).

GSK-3 is the most commonly proposed and, in AD research, best studied serine/threonine kinase. It is known to initiate and increase tau phosphorylation causing a reduction in microtubule bundling (Wagner et al. 1996). Our results show that inhibition of tau phosphorylation by the GSK-3 inhibitor AR-A014418 caused decreased phosphorylation at residues Thr231 and Ser396 but only a weak reduction at Thr181 in SH-SY5Y-TMHT441 cells. These results are in accordance with previous data showing the involvement of GSK-3 in the activation of these and many other tau phosphorylation sites (Hanger and Noble 2011). In AD brains, it was demonstrated that GSK-3 initially accumulates in the cytoplasm of pre-tangle neurons (Pei et al. 1999) supporting *in vitro* data showing microtubule bundling in mammalian cells caused by GSK-3-dependent tau phosphorylation (Sang et al. 2001). Furthermore, over-expression of GSK-3 in an inducible transgenic GSK-3β mouse model causes learning deficits that were reversible after suppression of GSK-3β expression (Hernandez et al. 2002; Engel et al. 2006), suggesting that activation of tau phosphorylation by GSK-3 not only initiates neuronal pathology by boosting aggregate formation but actually affects memory skills. This is entirely consistent with more recent studies showing soluble hyperphosphorylated tau disrupting normal synapse function and memory function in *Drosophila* and murine models of AD (Cowan et al. 2010; Hoover et al. 2010).

Inhibition of tau phosphorylation by the CDK1/5 inhibitor RP106 (Aloisine) caused decreased phosphorylation at residues Thr181 and Ser396 but had no effect on Thr231 in SH-SY5Y-TMHT441 cells. Previous studies observed phosphorylation of all three residues by CDK1/5, but we were unable to see this reproducibly in the SH-SY5Y-TMHT441 cell line using Mesoscale Discovery assays (Liao et al. 2004; Hanger et al. 2007) (for a detailed list see http://cnr.iop.kcl.ac.uk/hangerlab/tautable). This discrepancy might depend on the use of different site-specific antibodies or TMHT mutation-specific conformational changes of tau. The CDK1/5 protein kinase is also known to be involved in microtubule assembly and disassembly (Baumann et al. 1993), reducing the ability of tau to associate with microtubules and therefore causing cytoskeletal disruption and morphological degeneration followed by apoptosis (Patrick et al. 1999). In mice over-expressing the CDK5 activator p25, aggregated tau accumulates in the brainstem and cortex leading to an increased number of neurofibrillary tangles (Noble et al. 2003). Injection of CDK5 in the rat hippocampus caused hyperphosphorylation of tau at different phosphorylation sites, among others Ser396/404, leading to an impaired spatial memory of the animals (Liao et al. 2004). These results fill the gap between *in vitro* and *in vivo* data of patients with AD, where the CDK5 activator p25 was shown to be up-regulated in the brain (Patrick et al. 1999).

GSK-3β and CDK5 are also known to be key kinases in amyloid β-driven tau phosphorylation (Blurton-Jones and Laferla 2006). Cytotoxic amyloid β accumulates intraneuronally in the brain of AD patients and APP phosphorylation is suggested to play an important role in its amyloidogenic processing. Specifically amyloid β42 is shown to induce tau phosphorylation and formation of neurofibrillary tangles via the GSK-3 and CDK5 pathway in both *in vitro* and *in vivo* studies (Takashima et al. 1998; Town et al. 2002; Otth et al. 2002; Gotz et al. 2001). Amyloid β-induced toxicity seems to depend on considerable levels of tau (Liu et al. 2004) that can be inhibited by treatment with the neuroprotective peptide davunetide or GSK-3 inhibitors (Matsuoka et al. 2007; Medina and Avila 2010). Davunetide and the GSK-3 inhibitor tideglusib are currently in phase II clinical trial for the treatment of predicted tauopathies or AD and PSP, respectively (ClinicalTrial.gov identifier-davunetide = NCT01056965, tideglusib = NCT01049399 and NCT01350363), providing first clinical data about the efficiency of tau phosphorylation modulating drugs (Gozes 2010). Therefore, treatment of the here presented SH-SY5Y-TMHT441 cell line with amyloid β peptide might provide an appropriate model for amyloid β-driven tau phosphorylation.

Inhibition of tau phosphorylation by the CK1 inhibitor IC261 caused decreased phosphorylation at residues Thr231 and Ser396 but not at Thr181 in SH-SY5Y-TMHT441 cells. Until now, it was only shown that CK1 can phosphorylate tau at residue Ser396, but not at Thr231 (Hanger et al. 2007). However, CK1 kinase often acts as a modulator of GSK-3 phosphorylation by prephosphorylating tau, resulting in a stronger phosphorylation of the same phosphorylation site by GSK-3 (Singh et al. 1995), supporting our data that CK1 and GSK-3 inhibitors suppress the same sites. It was previously shown that CK1 phosphorylation of tau initiates a decrease in the fraction of bundled tau bound to microtubules and that tau residue Ser396 is one of the essential phosphorylation sites for this event (Li et al. 2004). Additionally, it was shown that CK1 is over-expressed at both protein and mRNA levels in AD brain tissue (Ghoshal et al. 1999; Yasojima et al. 2000) and that CK1 in human tissue associates with paired helical filaments (Kuret et al. 1997), making this phosphorylation site disease specific.

Since the IC261 inhibitor is known to act on CK1 isoform CK1δ and CK1ε and at higher concentrations also on CK1α1, at least one of these isoforms has to be activated in SH-SY5Y-TMHT441 cells (Mashhoon et al. 2000). To further understand the role of CK1 and more specifically CK1δ in the phosphorylation of tau, a SRM-based assay that measures phosphorylation at tau sites, known to be exclusively phosphorylated by these kinases, is currently under development.

Since it is known from this and other studies that several kinases can act on the same phosphorylation site (Hanger et al. 2009), a complete inhibition of phosphorylation at a single site by using only one specific kinase inhibitor seems not possible. In this study, relatively high concentrations of the inhibitors were used to reach the maximum effect for every single kinase on different phosphorylation sites. Therefore, dose–response curves displayed not the full spectrum and IC_50_ could be determined only approximately by non-linear regression. Calculations were possible for SP600125-Thr181, SP600125-Thr231, AR-A014418-Thr231, and IC261-Thr231. A complete sigmoidal dose–response curve could only be generated for AR-A014418-Thr231 showing only a weak maximal inhibition of about 25%. An interesting approach for future studies would be a co-application of two or more kinase inhibitors. This strategy might give an insight in the number of kinases involved in the phosphorylation of a single tau phosphorylation site and the contribution of every single kinase.

By using the specific kinase inhibitors SP600125, AR-A014418, RP106, and IC261 (Bennett et al. 2001; Mettey et al. 2003; Mudher et al. 2004) we were able to provide an insight into the protein kinases involved in the phosphorylation of SH-SY5Y-TMHT441 cells. The observed effects demonstrate that SH-SY5Y-TMHT441 cells largely reflect the phosphorylation events observed in human diseased brain tissue (Wray et al. 2008). The tau over-expressing cell line, SH-SY5Y-TMHT441, clearly reflects the complexity of tau hyperphosphorylation seen in human tauopathies as evidenced by selective inhibition of site-specific phosphorylation by four well-known tau kinases. We assume that these cells also express other kinases including PKA, ERK1/2, p38MAPK, PKC, and many more (for a detailed list see http://cnr.iop.kcl.ac.uk/hangerlab/tautable) involved in tau phosphorylation events *in vivo* and, therefore, represent a suitable cell culture system for the fast screen of novel phosphorylation modifying compounds directed against tau hyperphosphorylation, building the foundation for follow-up *in vivo* proof-of-concept studies in TMHT mice that express virtually the same gene construct.

Combined analyses of new tau-phosphorylation modifying drugs in the SH-SY5Y-TMHT441 cell line and the TMHT mouse model will enable fast and reliable *in vitro* and *in vivo* examinations of drug efficacy.
